# Supplementary material for: SpAD Biofunctionalized Cellulose Acetate Scaffolds Inhibit Staphylococcus aureus Adherence in a Coordinating Function with the von Willebrand A1 Domain (vWF A1)
Source: J Funct Biomater. 2022 Feb 21;13(1):21. doi: 10.3390/jfb13010021 (PMC8883972; doi:10.3390/jfb13010021)
Supplement: Supplementary file 1 [file jfb-13-00021-s001.zip › jfb-1510673-supplementary.pdf]

Journal of
Functional Biomaterials

MDPI

Article

SpAD Biofunctionalized Cellulose Acetate Scaffolds Inhibit *Staphylococcus aureus* Adherence in a Coordinating Function with the von Willebrand A1 Domain (vWF A1)

Stefanos Pendas [1,2], Antonis Asiminas[3], Alexandros Katranidis[4], Costas Tsioptsias [5], Maria Pitou [1], Georgios Papadopoulos [6] and Theodora Choli-Papadopoulou [1,*]

[1] Laboratory of Biochemistry, Department of Chemistry, Aristotle University of Thessaloniki, University Campus, 54124 Thessaloniki, Greece; spidy1927@yahoo.gr (S.P.); margeopit@chem.auth.gr (M.P.)
[2] General Hospital of Thessaloniki "St. Demetrios", Elenis Zografou 2, 54634 Thessaloniki, Greece
[3] Division of Glial Disease and Therapeutics, Center for Translational Neuromedicine, University of Copenhagen, Nørre Alle 14, 24.3.9, 2200 Copenhagen N, Denmark; a.asiminas@sund.ku.dk
[4] Institute of Biological Information Processing IBI-6, Forschungszentrum Jülich (FZJ), 52425 Jülich, Germany; a.katranidis@fz-juelich.de
[5] Department of Chemical Engineering, Aristotle University of Thessaloniki, University Campus, 54124 Thessaloniki, Greece, ktsiopts@gmail.com
[6] Department of Biochemistry and Biotechnology, University of Thessaly, Biopolis, 41500 Larissa, Greece; geopap@bio.uth.gr
* Correspondence: tcholi@chem.auth.gr

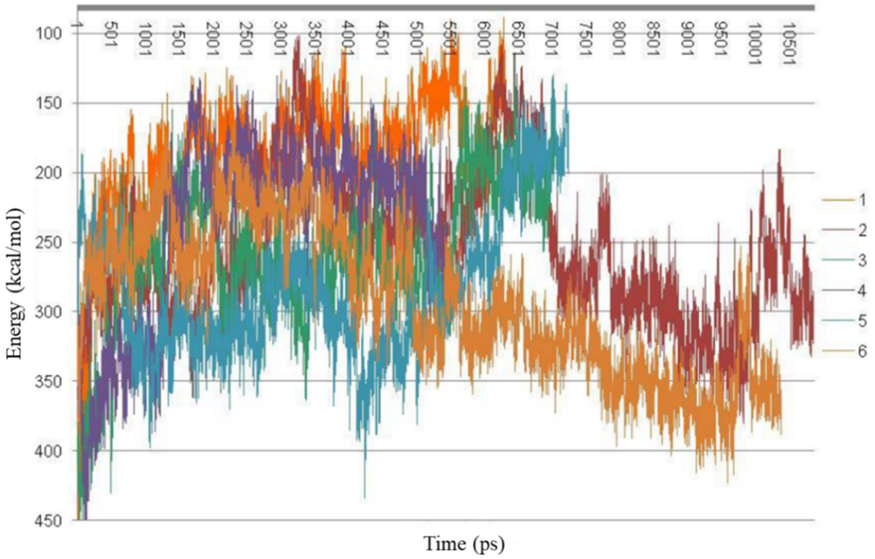

Figure S1. Comparison of binding energies for complexes 1-6. The low energy value for complex 6, which is also the most likely conformation for the interaction, is evident. The value of the final energy is very close to the energy provided by the zdock server (-370 kcal/mol).
